# Timing of Revascularization and Parenteral Antibiotic Treatment Associated with Therapeutic Failures in Ischemic Diabetic Foot Infections

**DOI:** 10.3390/antibiotics12040685

**Published:** 2023-03-31

**Authors:** Dominique Altmann, Felix W. A. Waibel, Gabor Forgo, Alexandru Grigorean, Benjamin A. Lipsky, Ilker Uçkay, Madlaina Schöni

**Affiliations:** 1 Balgrist University Hospital, University of Zurich, 8008 Zurich, Switzerland; 2 Department of Orthopedics, Balgrist University Hospital, University of Zurich, 8008 Zurich, Switzerland; 3 Department of Angiology, University Hospital Zurich, University of Zurich, 8091 Zurich, Switzerland; 4 Department of Medicine, University of Washington, Seattle, WA 98195-6420, USA; 5 Unit for Clinical and Applied Research, Infectiology, Balgrist University Hospital, University of Zurich, 8008 Zurich, Switzerland

**Keywords:** parenteral antibiotic therapy, limb ischemia, diabetic foot infection, peripheral arterial disease, timing of angioplasty, clinical and microbiological failures

## Abstract

For ischemic diabetic foot infections (DFIs), revascularization ideally occurs before surgery, while a parenteral antibiotic treatment could be more efficacious than oral agents. In our tertiary center, we investigated the effects of the sequence between revascularization and surgery (emphasizing the perioperative period of 2 weeks before and after surgery), and the influence of administering parenteral antibiotic therapy on the outcomes of DFIs. Among 838 ischemic DFIs with moderate-to-severe symptomatic peripheral arterial disease, we revascularized 608 (72%; 562 angioplasties, 62 vascular surgeries) and surgically debrided all. The median length of postsurgical antibiotic therapy was 21 days (given parenterally for the initial 7 days). The median time delay between revascularization and debridement surgery was 7 days. During the long-term follow-up, treatment failed and required reoperation in 182 DFI episodes (30%). By multivariate Cox regression analyses, neither a delay between surgery and angioplasty (hazard ratio 1.0, 95% confidence interval 1.0–1.0), nor the postsurgical sequence of angioplasty (HR 0.9, 95% CI 0.5–1.8), nor long-duration parenteral antibiotic therapy (HR 1.0, 95% CI 0.9–1.1) prevented failures. Our results might indicate the feasibility of a more practical approach to ischemic DFIs in terms of timing of vascularization and more oral antibiotic use.

## 1. Introduction

The prevalence of diabetes mellitus, and its complications, is rising worldwide [1], and one of its major consequences is foot complications. These are frequently associated with diabetic foot ulcers (DFUs) that ultimately may become diabetic foot infections (DFIs) [1,2,3]. Of all DFIs, 17% will require at least one or more lower extremity amputations during the patient’s lifetime [3]. These amputations are required not only because of infections, but also other complicating factors. Most below-knee amputations are predominantly necessitated by the presence of peripheral arterial disease related ischemia, whereas toe amputations are more often necessitated by factors such as destructive bone lesions, hammer toe deformities, and the presence of refractory DFUs, which can be further complicated by acute ischemia [1,2,3,4,5,6]. While appropriately administered medical and surgical therapy can often cure DFIs, clinical failures during or after treatment can be as high as 25%, even in specialized centers in resource-rich settings [5,6,7]. Reasons for these clinical failures are often complex and may be caused by the presence of multiple and simultaneous clinical entities [8,9,10,11]. Among these, foot ischemia usually plays the predominant role. Among all clinical failures, only 5–15% are true infection recurrences, which have previously been called the microbiological failure [5,6]. This is a subset of clinical failure in which the majority of the causative pathogens in the recurrence are identical to those of the prior infection [5,7]. This relatively infrequent type of infectious recurrence is most often attributed to poor patient compliance or clinicians selecting the wrong empirical antimicrobial therapy. There is an additional entity deemed “persistent infection” that may occur during the second or third week of a (targeted) antibiotic therapy. In our own clinical experience, these persistent infections are actually more often related to aggravating ischemia, poor patient adherence, or new pathogens selected by the antibiotic therapy in use [12,13].

Caring for a patient with an ischemic diabetic foot optimally requires a multidisciplinary team and dedicated surveillance to prevent further complications [4]. The occurrence of infection is most often the ultimate and limb-threatening complication of chronic underlying ischemic or neuropathic problems that have remained unresolved. DFIs can be devastating, especially amputations, but also lead to prolonged hospital stays, long-duration antibiotic therapy [5], and adverse events related to therapies [5,6]. In several prospective trials, antibiotic-related adverse events occurred in 10–15% per DFI episode [5,6,14,15]. Long-term administration of antibiotics by the intravenous route usually yields a higher burden of adverse events. While sharing the known possible antibiotic-related complications, parenteral therapies have the additional potential burden of nondrug-related problems, e.g., higher costs, difficulties arranging for administration during weekends, catheter-related complications [16], and increased sodium load potentially contributing to the frequent risk of postoperative heart decompensations [17,18].

Many clinicians consider clinically significant peripheral artery disease (PAD) a particularly concerning risk for therapeutic failure in patients with DFIs [19,20]. The reduced blood supply impairs the wound healing [21] as well as the delivery of antibiotics to the infected site. We have found that in DFIs the likelihood of both clinical failure (hazard ratio (HR) 6.1) and major amputation (HR 8.0) is significantly associated with the advanced PAD [7]. This leads many clinicians to view the presence of severe PAD as an indication for prolonged initial intravenous antibiotic therapy in DFI, despite the associated increased costs, and impaired postsurgical mobilization [18]. However, practically all available published retrospective trials have failed to show any protective effect on clinical failure in DFIs with prolonged parenteral antimicrobial medication, including β-lactam agents [5,6,22,23]. 

In adult diabetic patients, PAD is usually associated with an infrageniculate pattern of severity [8,24,25]. The prevalence of PAD in elderly diabetic patients is two- to sevenfold higher than in similar nondiabetic patients [26], and at least half of the patients with a chronic DFU have concurrent PAD [8,27]. In patients with ischemic diabetic foot wounds, revascularization may reduce the risk of limb loss and improve wound healing [9,28,29,30,31], but amputation remains a distinct possibility despite one or several attempts of revascularization [19,32]. 

The continuing problem with clinical failures in DFIs raises the question of what might be the optimal timing of any required revascularization. There is a widespread belief that it is imperative that revascularization should be undertaken before debridement surgery, with an aim to maximize the chances for rapid and definitive wound healing. Similarly, because time is of the essence in preserving ischemic tissue, many experts further believe that a successful intervention should occur immediately before surgery. Such a fixed sequence of consecutive interventions in a short time interval, however, causes major logistical problems. Even in resource-rich settings [19], it is not common for hospitals to have dedicated, specialized staff, and always-available facilities to allow for affordable and timely revascularization [19]. For this reason, adherence to a fixed scheduled sequence might actually become counterproductive to reducing clinical failures.

With these issues in mind, we designed this large retrospective, single-center, case–control study of patients with moderate-to-severe ischemic DFI to investigate the interactions among therapy with parenteral antibiotic regimens, the sequence and the timing between surgery and vascularization, and the clinical influence of a delayed angioplasty in surgically operated DFI patients.

## 2. Results

### 2.1. Study Populations

Our DFI registry over 20 years identified a total of 838 ischemic DFI episodes in adult patients [33], with a large case-mix of various concomitant co-morbidities. Table 1 displays the most pertinent patient characteristics of the entire cohort and the subset of 608 ischemic DFI patients who underwent lower extremity revascularization (98 females, 16.1%; median age 69.5 years). The median clinical follow-up time was 6.7 years for the entire cohort, and 6.4 years for the revascularized group. The duration of active smoking was 40 pack-years. For the rest of the analyses, we concentrated on just the revascularized patients in an attempt to homogenize our study population. 

### 2.2. Angiologic Interventions

The degree of PAD was moderate to severe in most cases, as defined by the latest IWGDF criteria [4]. The largest group of DFI episodes was associated with a PAD grade of II (n = 237; 39%) [34,35], followed by grade IV (168; 28%), grade I (137; 23%), grade III (6; 1%), and an undetermined grading (10%). The most frequent PAD localization was crural (40.8%). In a more detailed examination, a 2-vessel runoff represented 16.7% of the lower leg PADs with a median ankle–brachial index (ABI) of 0.8, a median toe systolic pressure of 70 mmHg, and the absence of palpable pedal pulses in 77.1% of cases. Angiographically, a multi-level pattern of substantial stenosis (44.2%) was predominant in revascularized patients. Demographic parameters were similar in the DFI groups with or without revascularization (Table 1).

Of the 608 patients with ischemic DFIs, 61% (373/608) were revascularized within a few days before surgery. Among those who were revascularized after surgery, 28 had the procedure immediately after surgery, as planned. The rest were revascularized days or weeks after their debridement surgery. The median delay between revascularization and debridement surgery was 7 days (range, 0–14 days), with the most urgent cases usually operated on within 3 or 4 days. In the immediate aftermath of revascularization by angiography, the angiologists in charge deemed 78% of their interventions satisfactory. We performed only one revascularization attempt before surgery. If the patient needed additional revascularization, further angioplasties were performed after the surgical debridement or even after hospitalization. The median length of hospital stay in our orthopedic wards was 17 days.

### 2.3. Infections and Antibiotic Treatments

Culture of the intraoperative tissues (121 entirely soft tissue and 487 episodes with bone specimens as well) yielded 207 different microbiological constellations. The three represented pathogen groups were *Staphylococcus aureus*, Gram negatives (including Gram-negative anaerobes), and streptococci. Infection was classified by the IDSA/IWGDF criteria as moderate or severe [4] for the majority of cases. The median serum C-reactive protein level at admission was 72 mg/L.

Before the intraoperative microbiological samplings, 124 (20%) of the DFI cases had already received (empirical) antibiotic therapy, with a median duration of 2 days prior to admission (range, 1–270 days). Overall, the patients in this study were treated with 164 different antibiotic regimens, of which 171 were empirical. The three most frequent parenteral antibiotic agents used were co-amoxiclav (302 episodes; 50%), piperacillin–tazobactam (45%), and vancomycin (11%). The median total duration of postoperative antibiotic therapy was 21 days. The overall median duration of parenteral antibiotic therapy, which always started during the surgical debridement in the operating room, was 7 days. It ranged from 1 to 103 days (interquartile range, 4–14 days). In detail, the patient group with successful revascularization received a slightly shorter duration of parenteral antibiotic treatment than those who were clinical failures (median 8 versus 7 days; *p* = 0.03), but this small difference was statistically significant (Table 1). 

**Figure 1:**
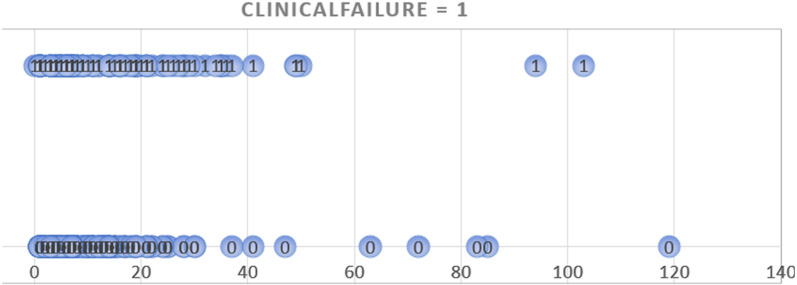
Graphic plotting of the duration of parenteral antibiotic treatment (horizontal axis; in days) against the occurrence of clinical failure (circles with the number 1) or remissions (circles with the number 0). The bulk of both, failures and remissions, situates in the left part of the figure, i.e., within a few days of parenteral therapy.

The decisions for administering parenteral antibiotics were made by the clinicians in charge of the patients, especially taking into consideration the presence of (presumed) bacteremia. In only 15 DFI cases (2.4%) the antibiotic therapy was administered intravenously for a prolonged period due to a lack of appropriate oral administration possibilities. We plotted the number of intravenous antibiotic days against the incidence of remission and clinical failures and did not detect a minimal threshold that would be protective against failures (Figure 1). We also followed up the DFI patients after discharge and did not see evidence that they received additional, or new, antibiotic treatments elsewhere. Only in cases of clinical failures other physicians, usually the general practitioner, did reinstall an oral antibiotic treatment before contacting us. During the study period, we did not administer any local (intraosseous) antibiotic formulations [36]. Unfortunately, based on the records, we were unable to (retrospectively) evaluate the detailed costs of the various antibiotic regimens used by these patients.

### 2.4. Clinical and Microbiological Failures

Among the 608 revascularized DFI episodes, there were 182 clinical failures (29.9%) and 31 (5.1%) microbiological failures. The majority of patients needed only a single surgical revision. However, these revisions were substantial, including a major lower extremity amputation in 98 episodes (16.1%). Table 2 compares patients with revascularized DFIs who achieved “remission” (successful treatment of infection) versus those with clinical failures.

### 2.5. Multivariate Adjustments

The case-mix of the study population remained large, even if we homogenized them to only DFI cases with revascularization. To adjust for the case-mix, we performed multivariate analyses, rather than further stratifications. The results of our univariate and multivariate Cox regression analyses of factors related to clinical failure are displayed in Table 3 and related to microbiological failure in Table 4. The time delay between surgery and the revascularization (hazard ratio (HR) 1.0, 95% confidence interval (CI) 1.0–1.0), or a postsurgical timing (HR 0.9, 95% CI 0.5–1.8) was not associated with the outcome clinical failure. Only the angioplasty of the Arteria poplitea (HR 2.3, 95% CI 1.2–4.6) was associated with clinical failure. In the multivariate adjustment based on parenteral antibiotic use, neither the total duration of antibiotic therapy (HR 1.0; 95% CI 1.0–1.0; Table 3), nor the duration of parenteral administration (HR 1.0; 95% CI 0.9–1.1; Table 4) was associated with clinical failure. The results were also similar for “microbiological failure” with the corresponding HR 1.0, 95% CI 1.0–1.0 and HR 1.0, 95% CI 0.8–1.1, respectively. 

## 3. Discussion

In our case–control study of 608 adult patients revascularized as part of their treatment for DFIs, we did not detect any angiologic or antibiotic-related parameters that were significantly associated with the occurrence of either clinical or microbiological failures other than angioplasty of the popliteal artery. This latter finding is likely to be a coincidence. None of the vascular parameters, such as the PAD localization, the PAD severity, the PAD type, the runoff type, and the residually patent vessels in 2- or 1-vessel runoff configurations, were associated with the risk of failures. Therefore, we found no confirmation for the common belief that for ischemic DFIs, revascularization should occur before any required orthopedic surgery. Lastly, we also did not find evidence for an ideal time point to undertake revascularization. In the literature, the need for urgent surgery largely depends on the patient’s ischemic symptoms [32]. Some research groups suggest that undertaking early vascular interventions could increase the healing chances, especially in diabetic adults [8,19,20,30]. Others are less strict, advocating that patients benefitting from revascularization should be selected carefully, including considering the comorbidities, chances of success, and the likelihood of recovery from the infection [31,37,38].

At the first glance, our findings reflect the insidious nature of both PAD and diabetes. Among all the major risk factors for developing PAD, only stopping of smoking, achieving better glycemic control, and perhaps weight reduction are realistic possibilities for prevention. Similarly, efforts to prevent foot infection, e.g., by good foot hygiene, avoiding foot ulceration, and possibly glycemic control, probably only have limited success. As with PAD, smoking is a substantial risk for poor wound healing in ischemic tissues and appears to be an independent risk factor for developing infections in surgical sites after orthopedic surgery [39]. While helping patients to stop smoking is a difficult challenge, doing so for a brief period of time when the patient has a DFI or requires elective foot could be highly beneficial. 

A second important message from our findings is that contrary to common belief, the duration of initial parenteral antibiotic administration was not associated with the risk of clinical or microbiological failure. This result is in line with all our own retrospective analyses, as well as other published literature [5,6,22]. These studies generally failed to determine a minimal duration of intravenous administration that significantly improved the fate of the ischemic and infected foot. The highly useful OVIVA trial, which prospectively randomized patients with musculoskeletal infections to parenteral antibiotic treatment for only 7 days, versus a much longer course, found no difference in clinical outcomes [16]. A subanalysis of this landmark trial examining the patients with diabetic foot osteomyelitis equally failed to demonstrate any significant differences between those treated with predominantly intravenous antibiotic therapy compared to those treated with predominantly oral antibiotics, it did not specifically target DFI patients with PAD [40].

The existing literature on the questions we addressed in this study is large, sometimes contradictory, and addressed several variables beyond those we targeted. For instance, according to guidelines of the International Working Group on the Diabetic Foot (IWGDF), the presence of a skin perfusion pressure (tcPO2) of ≥40 mmHg, a toe pressure of ≥30 mmHg, or a tcPO2 of ≥25 mmHg increases the probability of a diabetic foot wound healing by 25% [8]. Another study of patients with a DFU found that a tcPO2 of <46 mmHg is associated with ulcer recurrence, and that an ankle–brachial index of <0.9 predicted a higher risk of a secondary minor amputation [29]. On the other hand, a large retrospective study from Geneva, Switzerland, failed to find any specific tcPO2 cutoffs per se (especially not in the interval between 20 and 40 mmHg) that predicted the risk of postsurgical wound failures [34,35]. The measurement of the tcPO2 is only one of many cornerstones in the presurgical assessment of PAD in a patient presenting with a DFI and should not be interpreted alone as having absolute predictive value [9,32,41]. 

As with PAD, infection of the foot in diabetic patients is related to a panoply of other underlying problems, including patient malcompliance with foot care, limb ischemia, peripheral and polyneuropathy, poor wound care, and off-loading [42]. Both PAD and infection also complicate the approach to required surgical interventions. With so many underlying, and dynamic, variables, no single therapeutic parameter will likely change the ultimate outcome. We can usually heal a specific DFI episode, but if the reasons are not adequately addressed the patient will likely face another [12,43].

Our finding of a failure to demonstrate the importance of the revascularization sequence, or of the necessity for intravenous antibiotic therapy, can increase streamlining the management of DFI patients. We believe these data suggest that vascular interventions can be scheduled more flexibly, with the timing adjusted with attention to symptoms, practicability, and organizational issues. It is much more important to do the revascularization than to obsess over a sequence of this procedure versus other surgical treatments. We believe the main message of our study is that it is not necessarily inappropriate to decide to revascularize after any required orthopedic interventions or to decide against routine intravenous antibiotic therapy in ischemic DFIs [44]. 

### 3.1. Limitations

While our study benefits from investigating a single-center cohort with over 600 ischemic DFI episodes, our study also has limitations. First, it has the inherent limitations of a retrospective design. For example, DFI episodes for which treatment ultimately failed were associated with a strikingly longer duration of total antibiotic therapy (including that administered intravenously) than cases with ultimate remissions. This is a classical “confounding by indication” that plagues retrospective case–control studies that investigate multidisciplinary pathologies. Furthermore, we were unable to assess the timing of revascularization as it could be completed in a prospective randomized trial, for organizational and ethical reasons. The question of the value of intravenous versus oral antibiotic therapy can best be addressed by a randomized control trial, which we think would likely confirm the noninferiority found in the OVIVA trial [16,40]. Secondly, our cohort of patients was very heterogeneous, which is inherent to trials in a DFI population. Thirdly, our departments of orthopedic surgery and angiology are separated by three kilometers. It could theoretically be that our measured delays would be different, if both interventions were available in the same building. So far, quality interventions for the diabetic foot target the availability of rapid and easily accessible revascularization facilities, rather than to establish a geographical proximity between the different departments [19]. Fourthly, our DFI patients may have been treated elsewhere with antibiotic agents after the hospitalization in our clinic without our knowledge, especially with oral antibiotics. As we regularly followed up all hospitalized patients in our outpatient clinic, we consider the possibility of unreported intravenous antibiotic therapy to be minimal. Finally, we assessed many clinical variables and placed our emphasis on established clinical parameters regarding the management of DFI. This list was far from being exhaustive and lacks potentially interesting variables under current investigation, such as high-intensity statin therapy [42], different angioplasty technologies, or professional nutritional interventions for wound healing in the diabetic foot [33]. 

### 3.2. Conclusions

In our retrospective, single-center cohort study, we failed to show that the sequence, or the timing, of revascularization alters the clinical or microbiological outcomes of ischemic DFIs in patients who undergo orthopedic debridement. We found no evidence that prolonged parenteral administration of antibiotics offered any benefit compared to oral antibiotic agents. Using the information from our findings might lead to a more streamlined approach in terms of the timing of revascularization and antibiotic therapy.

## 4. Methods

### 4.1. Setting and Management of Diabetic Foot Infections

The Balgrist University Hospital is a tertiary orthopedic clinic that cooperates with the Department of Angiology at the “University Hospital Zurich”. The Diabetic Foot Unit runs several registers regarding DFIs, assessing episodes since 1 January 2000 [5,7,33,35]. The first, bedside angiologic examinations (ABI, pressures, pulse palpability) are initiated by the specialized orthopedic surgeons of the Diabetic Foot Unit. The angiologists confirm the indication for revascularization or angiography and organize the scheduling of the interventions. All of our specialized orthopedic surgeons, internists, and infectious diseases physicians caring for patients have a long experience in the management of diabetic foot problems [44]. Since 2018, all of them are committed to an “antibiotic stewardship approach” to DFI management [44], by conducting randomized-controlled trials on DFIs [15], and by prescribing antibiotic drugs as infrequently, for as short duration, and with the narrowest spectrum as possible. We also aim for an early switch from intravenous to oral treatment, to withhold empiric antibiotic use before the intraoperative tissue sampling for microbiology and to avoid expensive or toxic drugs [44]. Of course, there are exceptions to these general recommendations. For instance, in cases with a rapidly spreading soft tissue DFI, or when concomitant bacteremia is suspected, we start antibiotic therapy directly after admission, with parenteral therapy and before performing the intraoperative tissue samples. 

A particular feature of our medical center concerns the modalities of the patients’ transport between buildings. Patients receiving only oral medication are transported in a sitting position without medical surveillance. In Zurich, this transfer roughly costs 35 Swiss Francs (equaling 37 $US). If the same patient is receiving parenteral medication, the transport occurs in a supine position with an ambulance, which costs 220 to 350 Swiss Francs. Hence, the transfer costs can increase up to nine times only because of the presence of a peripheral venous catheter for parenteral antibiotic use. 

### 4.2. Study Population, Follow-Ups, and Definitions

For this retrospective case–control cohort study, we included all adult DFI patients from 1 January 2000 to 31 December 2020, who had concomitant symptomatic PAD. We had three main study questions:

(a) Does the sequence of when revascularization and orthopedic (debridement) surgery are completed affect the likelihood of clinical or microbiological failure?
(b) Is the time interval between when revascularization and orthopedic (debridement) surgery are performed important (within a traditional time period of 2 weeks before and after surgery)?
(c) Is the duration of the initial parenteral antibiotic therapy related to the likelihood of remission of an ischemic DFI?

The confirmation of the presence of infection of the foot was based on the recommendations of the IWGDF guidelines and was supported by intraoperative microbiological tissue samples, imaging reports, and histology results (if available) [4]. We defined “remission” of infection as the absence of any clinical, laboratory, histologic, or imaging elements indicating a persistence of local infection. We defined “clinical failure” as the need for any unplanned revision surgery related to the index infection, but not any additional angiological intervention, without wound interventions or surgery. We defined “microbiological failure” as the recurrence of infection, after or during the combined medical and surgical therapy, with the same pathogens from an intraoperative specimen found in the index of DFI episode. We diagnosed PAD according to the Fontaine classification and relied on clinical confirmation after an endovascular procedure [24]. We assessed the following PAD parameters: PAD localization (pelvis, thigh, lower leg, multilevel, acral), pulse status (palpable pulses of *A. dorsalis pedis, A. tibialis posterior*), ankle–brachial index before and after revascularization, runoff type (3-vessel runoff, 2-vessel runoff, 1-vessel runoff, only collaterals), an obstructed artery in case of 2-vessel runoff (*A. tibialis posterior*, *A. tibialis anterior*, and *A. fibularis*), open vessels in case of a 1-vessel runoff (*A. tibialis posterior*, *A. tibialis anterior*, and *A. fibularis*). We completed our assessment by obtaining a transcutaneous oxygen pressure (tcPO2) and soliciting the past history of any revascularization. The angiologists defined the immediate success of their angioplasty by the residual and/or restent stenosis. The follow-up of the participants included both the regular, clinical follow-up at our diabetic foot clinic and the routine angiological controls. 

### 4.3. Statistical Analyses

The primary outcome was the risk of clinical failure within 12 months after the combined medical and surgical therapy for ischemic DFI. The secondary outcome was a microbiological failure. We used Pearson‘s chi^2^ test (for categorical variables) or the Wilcoxon rank-sum test (for continuous, nonparametric variables) for crude group comparisons. To adjust for the large case-mix, we ran separate Cox regression analyses with the respective outcomes “clinical failure” and “microbiological failure”. When analyzing the timing of revascularization, we computed the number of delay days as a continuous variable and as stratified variables. For stratification, we fitted the overall delay into four strata, which we arbitrarily set at the 25%, 50%, 75%, and 100% percentiles. We checked for collinearity and effect modification by interaction term and included 7–10 predictor variables per outcome variable in the final model. The timing of revascularization and the duration of parenteral antibiotic use were obligated parts of the final statistical models. The calendar date of the patient’s death, the date of the last medical control, or the occurrence of any failure defined the censor timepoint. Finally, we visually plotted the occurrence of clinical failure in relation to the number of effective parenteral antibiotic days. We used STATA^™^ software (Version 15, College Station, Texas, USA) and set the significance level (two-tailed) at 0.05.

## Figures and Tables

**Table 1:** Characteristics of the included patients with diabetic foot infections.

	Overall (n = 838; %)	Revascularized Patients (n = 608; %)
Age (median, in years)	69.3 (834)	69.5 (605)
Female sex	167 (19.9%)	98 (16.1%)
Body mass index (median; kg/m^2^)	29.6 (388)	29.7 (261)
Glycated hemoglobulin level at admission (%, median)	8 (44)	8 (35)
Glomerular filtration rate (mL/min/1.73 m^2^)	57 (472)	55 (341)
Smoking, pack-years (median; years)	37 (446)	40 (335)
Insulin treatment at admission No Yes	187 (22.3%) 651 (77.7%)	140 (23.0%) 468 (77.0%)
Renal dialysis at admission No Yes	750 (89.5%) 88 (10.5%)	544 (89.5%) 64 (10.5%)
Statin medication at admission No Yes	234 (32.6%) 484 (67.4%)	148 (28.9%) 364 (71.1%)
Total duration of antibiotic therapy (median; days)	20 days	21 days
Duration of intravenous antibiotic (median; days)	5 days	7 days
C-reactive protein level at admission (median; mg/L)	72.8 (179)	71.8 (123)
Peripheral arterial disease localization Pelvis Thigh Lower leg Multilevel Acral	9 (1.1%) 84 (10.0%) 342 (40.8%) 294 (35.1%) 2 (0.2%)	6 (1.0%) 58 (9.5%) 242 (39.8%) 269 (44.2%) NA
Chronic anticoagulation No anticoagulation No documentation Acetylsalicylic acid Clopidogrel Acetylsalicylic acid + Clopidogrel Phenprocoumon (coumarin) Rivaroxaban Apixaban Acetylsalicylic acid + Phenprocoumon Other combinations	62 (7.4%) 42 (5.0%) 360 (43.0%) 45 (5.4%) 93 (11.1%) 82 (9.8%) 5 (0.6%) 5 (0.6%) 25 (4.9%) 41 (4.9%)	26 (4.3%) 29 (4.8%) 255 (41.9%) 34 (5.6%) 85 (14.0%) 50 (8.2%) 1 (0.2%) 5 (0.8%) 16 (2.6%) 40 (6.6%)
Absent pedal pulses (median; SD)	646 (77.1%)	477 (78.5%)
Ankle–brachial index (median; SD)	0.8 (193)	0.8 (148)
Toe systolic pressure (median; SD)	70 (134)	63 (95)
Transcutaneous oxygen pressure (mmHg) Tibia Upper ankle joint Medial malleolus Lateral malleolus	49.5 (28) 41 (62) 43 (110) 42.5 (84)	49 (23) 40 (39) 40.5 (78) 42 (63)
Runoff type 3-vessel runoff 2-vessel runoff 1-vessel runoff Only collaterals	49 (5.9%) 140 (16.7%) 121 (14.4%) 12 (1.4%)	36 (5.9%) 105 (17.3%) 110 (19.1%) 10 (57.1%)
Obstructed vessel in case of 2-vessel runoff *A. tibialis posterior* *A. tibialis anterior* *A. fibularis*	82 (9.8%) 49 (5.9%) 9 (1.1%)	57 (9.4%) 43 (7.1%) 5 (0.8%)
Open vessel in case of 1-vessel runoff *A. fibularis* *A. tibialis anterior* *A. tibialis posterior*	62 (7.4%) 35 (4.2%) 22 (2.6%)	56 (9.2%) 31 (5.1%) 20 (3.3%)
In-stent stenosis No Yes	-	295 (87.5%) 42 (12.5%)
Angioplasty success No Yes	-	97 (23.5%) 316 (76.5%)
Angioplasty *A. femoralis superficialis* No Yes	620 (74.0%) 218 (26.0%)	-
Angioplasty *A. poplitea* No Yes	696 (83.1%) 142 (16.9%)	-
Angioplasty *A. tibialis anterior* No Yes	619 (73.7%) 219 (26.1%)	-
Angioplasty *A. tibialis posterior* No Yes	681 (81.3%) 157 (18.7%)	-
Angioplasty *A. fibularis* No Yes	664 (79.2%) 174 (20.8%)	-
Stratified delays between admission and angioplasty at 25% percentile at 50% percentile at 75% percentile at 100% percentile	-	104 (17.1%) 107 (17.6%) 164 (27.0%) 233 (38.3%)

**Table 2:** Comparison of patients with ischemic diabetic foot infections who had Clinical Failure versus Remission (n = 608) (including the multivariate results regarding the outcome “Clinical Failure”).

Variables (with Median Values)	Remission (n = 426)	Failure (n = 182)	*p* Value
Age	69.6 years	69.5 years	0.21
Glycated hemoglobulin level at admission	8%	8%	0.23
Ankle–brachial index	0.8	0.8	0.63
Delay between angioplasty and surgery	7 days	7 days	0.36
Surgery after angioplasty	259 (94%)	114 (90%)	0.18
Initial success of angioplasty	204 (78%)	86 (77%)	0.18
Receiving statin medication	260 (71%)	104 (70%)	0.80
Total duration of antibiotic use	20 days	28 days	***0.01***
Duration of parenteral antibiotic use	7 days	8 days	***0.03***

Statistically significant results are in italic and bold.

**Table 3:** Cox regression analysis of factors potentially associated with the outcome “clinical failure” (results expressed as hazard ratio with the corresponding 95% confidence interval).

Variables, n = 608	Univariate Results	Multivariate Results
Male sex	1.5 (1.0–2.3)	-
Age	1.0 (1.0–1.0)	1.0 (0.9–1.1)
Body mass index	1.0 (0.9–1.0)	-
Toe systolic pressure	1.0 (1.0–1.0)	1.0 (1.0–1.0)
Anticoagulation	1.1 (1.0–1.1)	0.3 (0.1–1.0)
Statin medication	1.4 (1.0–2.1)	-
Insulin	1.3 (0.9–1.9)	-
Renal dialysis	1.6 (1.0–2.5)	-
Antibiotic duration postoperative	1.0 (1.0–1.0)	1.0 (1.0–1.0)
Duration of parenteral antibiotic	1.0 (1.0–1.0)	1–0 (0.9–1.1)
Peripheral arterial disease (stages according to Fontaine) Stage 1 Stage 2 Stage 3 Stage 4 Stage unknown	Reference 1.4 (0.9–2.1) 0.4 (0.1–2.8) 1.4 (0.9–2.1) 1.1 (0.6–1.9)	-
Pulse (palpable) *A. dorsalis pedis* + *A. tibialis posterior* *A. dorsalis pedis* *A. tibialis posterior* No vessel	Reference 1.4 (0.7–3.1) 1.2 (0.4–3.1) 1.3 (0.7–2.4)	1.6 (0.6–4.2)
Run off type 3-vessel runoff 2-vessel runoff 1-vessel runoff Only collaterals	Reference 1.5 (0.7–3.1) 1.0 (0.5–2.0) 0.3 (0.1–1.4)	-
Ankle–brachial index	4.3 (0.8–23.9)	-
Delay revascularization (*continuous variable*)	1.0 (1.0–1.0)	1.0 (1.0–1.0)
Delay revascularization (*stratified variable*) 25% percentile of the overall delay 50% percentile 75% percentile	Reference 1.1 (0.6–1.7) 1.0 (0.7–1.6)	1.0 (1.0–1.0) 0.1 (0.0–9.7) 0.0 (0.0–8.3)
Angioplasty after surgery	0.9 (0.5–1.8)	1 (omitted)
Closed vessel in 2-vessel runoff *A. tibialis posterior* *A. tibialis anterior* *A. fibularis*	Reference 0.5 (0.2–1.0) 0.1 (0.0–2.3)	-
Run off 1	1.0 (0.6–1.6)	-
In-stent stenosis	0.7 (0.4–1.5)	-
Angioplasty *A. femoralis superficialis*	1.2 (0.9–1.7)	0.2 (0.0–5.1)
Angioplasty *A. poplitea*	1.2 (0.9–1.8)	-
Angioplasty *A. tibialis anterior*	1.2 (0.9–1.7)	-
Angioplasty *A. tibialis posterior*	1.5 (1.0–2.1)	-

**Table 4:** Univariate and multivariate analyses of factors potentially associated with the outcome “microbiological failure” *(results expressed as hazard ratio with the corresponding 95% confidence interval)*.

Variables, n = 608	Univariate Analysis	Multivariate Analysis
Male sex	6.6 (0.9–48.6)	-
Age	1.0 (1.0–1.1)	-
Body mass index	0.8 (0.7–1.0)	-
Smoking (pack-years)	1.0 (1.0–1.0)	1.0 (1.0–1.0)
Anticoagulation	1.0 (0.8–1.2)	-
Statin medication	2.6 (0.7–9.2)	-
Insulin	1.4 (0.5–3.8)	-
Dialysis	1.5 (0.5–4.3)	-
Antibiotic duration postoperative	1.0 (1.0–1.0)	1.0 (1.0–1.0)
Parenteral antibiotic duration	1.0 (1.0–1.0)	1.0 (0.8–1.1)
Peripheral arterial disease (stages according to Fontaine) Stage 1 Stage 2 Stage 3 Stage 4	Reference 1.3 (0.5–3.2) 0.4 (0.1–2.8) 0.8 (0.3–2.3)	-
Pulse (palpable) *A. dorsalis pedis* + *A. tibialis posterior* *A. dorsalis pedis* *A. tibialis posterior*	Reference 2.7 (0.2–30.1) 2.1 (0.1–34.1)	-
Toe systolic pressure	1.0 (1.0–1.0)	1.0 (1.0–1.0)
Run off type 3-vessel runoff 2-vessel runoff 1-vessel runoff Only collaterals	Reference 2.8 (0.3–22.4) 1.5 (0.2–12.7) ns	-
Ankle–brachial index	0.6 (0.4–1.0)	2.3 (0.6–9.4)
Angioplasty “entirely successful”	1.4 (0.5–4.1)	1.1 (0.1–11.4)
Delay revascularization (*continuous variable*)	1.0 (1.0–1.0)	1.0 (1.0–1.1)
Delay revascularization (*stratified variable*) 25% percentile 50% percentile 75% percentile	Reference 1.7 (0.5–5.9) 1.3 (0.4–4.2)	0.2 (0.0–32.6) 0.2 (0.0–33.0)
Angioplasty after amputation	1.2 (0.3–5.4)	1 (omitted)
Closed vessel in 2-vessel runoff *A. tibialis posterior* *A. tibialis anterior* *A. fibularis*	Reference 0.5 (0.1–2.4) 1.5 (0.2–14.1)	-
Run off 1	1.2 (0.4–3.5)	-
In-stent stenosis	0.4 (0.1–2.9)	-
Angioplasty *A. femoralis superficialis*	2.0 (1.0–4.2)	-
Angioplasty *A. poplitea*	1.6 (0.7–3.6)	-
Angioplasty *A. tibialis anterior*	2.0 (0.9–4.1)	-
Angioplasty *A. tibialis posterior*	1.5 (0.7–3.4)	-
Angioplasty *A. fibularis*	1.6 (0.7–3.4)	-

## Data Availability

We might provide anonymized key data upon reasonable scientific request to the corresponding author.
